# β-alanine suppresses malignant breast epithelial cell aggressiveness through alterations in metabolism and cellular acidity *in vitro*

**DOI:** 10.1186/1476-4598-13-14

**Published:** 2014-01-24

**Authors:** Roger A Vaughan, Nicholas P Gannon, Randi Garcia-Smith, Yamhilette Licon-Munoz, Miguel A Barberena, Marco Bisoffi, Kristina A Trujillo

**Affiliations:** 1 Department of Health, Exercise and Sports Science, University of New Mexico, Albuquerque, NM 87131, USA; 2 Department of Biochemistry and Molecular Biology, University of New Mexico Health Sciences Center, University Blvd, Albuquerque, NM 87131, USA; 3 Department of IFCE: Nutrition, University of New Mexico, Albuquerque, NM 87131, USA; 4 University of New Mexico Cancer Center, Albuquerque, NM 87131, USA; 5 Biological Sciences, Chapman University, Orange, CA 92866, USA

**Keywords:** Warburg metabolism, Glycolysis, Carnosine, Mitochondria, Oxidative metabolism, Proliferation, Migration, Doxorubicin

## Abstract

**Background:**

Deregulated energetics is a property of most cancer cells. This phenomenon, known as the Warburg Effect or aerobic glycolysis, is characterized by increased glucose uptake, lactate export and extracellular acidification, even in the presence of oxygen. β-alanine is a non-essential amino acid that has previously been shown to be metabolized into carnosine, which functions as an intracellular buffer. Because of this buffering capacity, we investigated the effects of β-alanine on the metabolic cancerous phenotype.

**Methods:**

Non-malignant MCF-10a and malignant MCF-7 breast epithelial cells were treated with β-alanine at 100 mM for 24 hours. Aerobic glycolysis was quantified by measuring extracellular acidification rate (ECAR) and oxidative metabolism was quantified by measuring oxygen consumption rate (OCR). mRNA of metabolism-related genes was quantified by qRT-PCR with corresponding protein expression quantified by immunoblotting, or by flow cytometry which was verified by confocal microscopy. Mitochondrial content was quantified using a mitochondria-specific dye and measured by flow cytometry.

**Results:**

Cells treated with β-alanine displayed significantly suppressed basal and peak ECAR (aerobic glycolysis), with simultaneous increase in glucose transporter 1 (GLUT1). Additionally, cells treated with β-alanine exhibited significantly reduced basal and peak OCR (oxidative metabolism), which was accompanied by reduction in mitochondrial content with subsequent suppression of genes which promote mitochondrial biosynthesis. Suppression of glycolytic and oxidative metabolism by β-alanine resulted in the reduction of total metabolic rate, although cell viability was not affected. Because β-alanine treatment reduces extracellular acidity, a constituent of the invasive microenvironment that promotes progression, we investigated the effect of β-alanine on breast cell viability and migration. β-alanine was shown to reduce both cell migration and proliferation without acting in a cytotoxic fashion. Moreover, β-alanine significantly increased malignant cell sensitivity to doxorubicin, suggesting a potential role as a co-therapeutic agent.

**Conclusion:**

Taken together, our results suggest that β-alanine may elicit several anti-tumor effects. Our observations support the need for further investigation into the mechanism(s) of action and specificity of β-alanine as a co-therapeutic agent in the treatment of breast tumors.

## Introduction

A multitude of factors influence cancer aggressiveness and invasiveness, many of which may be targeted by chemical agents as therapies [1]. Recent studies on cancer metabolism have revealed potential therapeutic targets that may slow cancer progression through restoration of a more normal metabolic phenotype [2-4]. Enhanced glucose uptake and aerobic glycolysis within tumors are well-document properties of tumor cell metabolism. Although increased glucose uptake has been exploited as a tool in early diagnostics such as in PET scan using ^18^F-deoxyglucose, heightened glycolytic reliance within tumor cells may provide numerous advantages to tumor cells [1,5].

One mechanism though which aerobic glycolysis may contribute to tumor aggressiveness is through acidification of the extracellular environment. For example, functional proteases are essential to tumor cell migration and invasion through tissue matrices. These proteases are dependent in great part on extracellular acidification, a direct function of cellular lactate export which is exacerbated in malignant phenotypes [6]. Furthermore, the acidic microenvironment of tumors can affect the efficacy of chemotherapeutic agents. For instance, the cellular uptake of Doxorubicin (Dox), an anthracycline which impedes tumor growth through DNA intercalation [7], is significantly affected by extra and intracellular pH. Dox acts as a weak base, and under protonated conditions is less able permeate to membranes [7,8]. Increased extracellular acidity such as that produced by aerobic glycolysis, reduces Dox uptake and alters cellular distribution leading to impaired efficacy [7,8]. Reduction of acidity has previously been shown to enhance both the uptake and toxicity of Dox in the malignant MCF-7 breast epithelial cell model [8].

β-alanine (3-aminopropanoic acid) is a non-essential amino acid found as a principal component of several commercially available sports supplements that purportedly increase athletic performance [9,10]. This is proposed to occur by the combination of β-alanine with histidine to form carnosine, which functions as an intracellular buffer [10]. Supplementation with β-alanine has been shown to delay accumulation of lactate during exercise through buffering the formation of lactate from pyruvate [10]. In cancer, the formation of carnosine is thought to be an under-explored agent with potentially therapeutic benefits [4]. Specifically, carnosine accumulation has been shown to reduce the proliferation of cultured of tumor cells and reduce tumor growth in vivo [4,11-13]. It is hypothesized that carnosine accumulation inhibits glycolysis, limiting energy production [4]. Additionally, carnosine has been shown to reduce cellular ATP production in glioma cells, and it is postulated that benefits of carnosine towards tumor inhibition are in part a result of glycolytic inhibition [4,14,15].

We demonstrate for the first time that treatment of breast epithelial cells with β-alanine reduces cancerous metabolism in addition to reducing extracellular acidification leading to suppressed aggressiveness. Furthermore, we demonstrate that β-alanine can increase the efficacy of Dox on MCF-7 cells at low concentrations.

## Methods

### Cell culture and treatments

Non-malignant breast epithelial cells (MCF-10a) and malignant breast epithelial cells (MCF-7) were purchased from ATCC (Manassas, VA). MCF-10a cells were cultured in combination F-12 DMEM media supplemented with 50 nM hydrocortisone, 20 ng/ml EGF, 0.01 mg/ml human insulin, 5% fetal bovine serum (FBS), and 1% Pen-Strep. MCF-7 cells were cultured in DMEM supplemented with 10% FBS with 0.01 mg/ml human insulin, and 1% Pen-Strep. Cells were treated with β-alanine from Sigma (St. Louis, MO) at 100 mM dissolved in media and incubated in 5% CO_2_ at 37°C for 24 hours.

### Metabolic assay

Cells were seeded overnight in 24-well culture plate from SeaHorse Bioscience (Billerica, MA) at density 5 × 10^5^ cells/well. To control for nitrogen load and hyper-osmolality, we also treated control cells with isonitrogenous valine or D-alanine. Following treatment, culture media was removed and replaced with XF Assay Media from SeaHorse Bioscience (Billerica, MA) containing 4500 mg/L glucose free of CO_2_ and incubated at 37°C. Per manufactures’ protocol, SeaHorse XF24 Extracellular Analyzer injection ports were loaded with oligomycin, an inhibitor of ATP synthase which induces maximal glycolytic metabolism and reveals endogenous proton leak (mitochondrial uncoupling) at a final concentration 1.0 μM. Measurement with oligomycin were taken for 24 minutes followed by the addition of carbonyl cyanide *p*-[trifluoromethoxy]-phenyl-hydrazone (FCCP), an uncoupler of electron transport that induces peak oxygen consumption (an indirect indicator of peak oxidative metabolism) at final concentration 1.25 μM. FCCP measurements were taken for 24 minutes followed by rotenone addition in 1.0 μM final concentration to reveal non-mitochondrial respiration and end the metabolic reactions [16,17]. Extracellular acidification rate (ECAR) calculated from proton production rate is an indirect measure of glycolytic capacity. Oxygen consumption (OCR), a measure of oxidative metabolism was measured using the SeaHorse XF24 Extracellular Analyzer from SeaHorse Bioscience (Billerica, MA). SeaHorse XF24 Extracellular Analyzer was run using 8 minute cyclic protocol commands (mix for 3 minutes, let stand 2 minutes, and measure for 3 minutes) as previously described [18,19].

### Cellular ATP content

Following treatment as described above, buffer-free media was added to the cells to measure total ATP production. The cells were lysed in 1% CHAPS lysis buffer from Chemicon (Billerica, MA) in PBS with Ca^2+^ and Mg^2+^ and the ATP-containing supernatant was recovered. Samples were allocated into a 96-well plate with a 1:1 dilution of ATP Bioluminescence Reagent from Sigma (St. Louis, MO) and ATP-containing samples (final reaction volume = 100 μl) and luminescence was measured as previously described [18].

### Quantitative real time polymerase chain reaction (qRT-PCR)

Following incubation, the total RNA was extracted using RNeasy Kit from Qiagen (Valencia, CA), per manufacturer’s protocol. Total RNA was quantified by Nanodrop spectrophotometry. cDNA was synthesized from 5000 ng total RNA using the Retroscript™ RT kit from Ambion (Austin, TX) according to manufacturer’s instructions. PCR primers were designed using Primer Express software from Invitrogen (Carlsbad, CA) and synthesized by Integrated DNA Technologies (Coralville, IA). Amplification of glucose transporter 1 (GLUT1), lactate dehydrogenase (LDH), and peroxisome proliferator-activated receptor gamma coactivator 1 alpha (PGC-1α) were normalized to the housekeeping gene, TATA Binding Protein (TBP). Table 1 summarizes the forward and reverse primers of each gene. qRT-PCR reactions were performed in triplicate using the LightCycler 480 real-time PCR system from Roche Applied Science, (Indianapolis, IN). SYBR Green based PCR was performed in triplicate using 5000 ng of cDNA per sample; final primer concentrations were 10 μM in a total volume of 30 μl. The following cycling parameters were used: 95°C for 10 minutes followed by 45 cycles of 95°C for 15 seconds, and 60°C for one minute. Relative expression levels were determined by the ΔΔCp method and compared to the lowest expressing group as previously described [18,20,21].

**Table 1:** Summary of qRT-PCR primers from integrated DNA technologies (Coralville, IA)

**Primer name**	**Forward sequence**	**Reverse sequence**
**GLUT1**	5′-ATCGTGGCCATCTTTGGCCTTT-3′	5′-CTGGAAGCACATGCCCACAAT-3′
**LDH**	5′-GCCCGTTTCCGTTACCTAAT-3′	5′- CTCCATAAGGGCACACTAGAATC-3′
**PGC-1α**	5′-ACCAAACCCACAGAGAACAG-3′	5′-GGGTCAGAGGAAGAGATAAAGTTG-3′
**TBP**	5′-CACGAACCACGGCACTGATT-3′	5′-TTTTCTTGCTGCCAGTCTGGAC-3′

*Abbreviations*: Glucose transporter 1 (GLUT1), lactate dehydrogenase (LDH), peroxisome proliferator-activated receptor gamma coactivator 1 alpha (PGC-1α), and TATA binding protein (TBP).

### Flow cytometry

Following treatment, the media was removed and the cells were re-suspended in pre-warmed media with 200 nM Mitotracker Green from Life Technologies (Carlsbad, CA) and incubated for 45 minutes as described above. The cells were pelleted, the media with Mitotracker was removed and the cells were suspended in pre-warmed media. Group mean fluorescence was measured using Facscalibur filtering 488 nm as previously performed [18]. To quantify protein expression, cells were treated as described before, trypsinized, permeabilized and blocked for 1 hour with PBS with 0.1% Triton 100X and 3.0% BSA from Sigma (St. Louis, MO). Cells were stained overnight with anti-GLUT1 antibodies (SPM498, mouse monoclonal) from Abcam (Cambridge, MA) at a final concentration of 1 μg/ml in PBS containing 0.1% BSA. The cells were rinsed with PBS with 0.1% Triton 100X and 3.0% BSA, and a secondary antibody (either AlexFluor 488 from Invitrogen (Carlsbad, CA)) was applied in 1:200 dilution for 1 hour [22]. Cells were then rinsed and re-suspended in PBS and the fluorescence was quantified as described above.

### Microscopy and immunocytochemistry

Chamber-slides from BD Bioscience (Sparks, MD), were seeded with 5000 cells/well. To verify protein expression, cells were cultured and treated for 24 hours as described above. Cells were fixed using 3.7% formaldehyde in media, permeabilized with PBS with 0.1% Triton 100X from Sigma (St. Louis, MO) for 10 minutes and blocked for 1 hour with PBS with 0.1% Triton 100X and 3.0% BSA from Sigma (St. Louis, MO). Cells were stained overnight with anti-GLUT1 antibodies (SPM498, mouse monoclonal) from Abcam (Cambridge, MA) at a final concentration of 1 μg/ml in PBS containing 0.1% BSA. The cells were rinsed with PBS with 0.1% Triton 100X and 3.0% BSA, and an AlexFluor 633 from Invitrogen (Carlsbad, CA) was applied in 1:200 dilution. Slides were mounted with Prolong Gold with DAPI from Invitrogen (Carlsbad, CA) and cured overnight. Cells were imaged using the Axiovert 25 microscope with AxioCam MRc from Zeiss (Thornwood, NY).

### Western blotting

Whole-cell lysates were collected following 24 hour treatment. Whole cell lysates were prepared by harvesting the cells on ice in high salt lysis buffer (25 mM Tris base, 8 mM MgCl2, 1 mM DTT, 15% glycerol, 0.1% Triton) supplemented with protease inhibitor mix (Sigma, St. Lois MO), followed by incubation on ice for 60 minutes. Insoluble material was removed by centrifugation at 17,500 × G for 3 minutes and protein concentrations were determined by Bradford assay (Protein Assay Dye Reagent Concentrate, Bio-Rad Laboratories, Hercules, CA). Total protein (120 μg per sample) was size-separated by 10% sodium dodecyl sulfate polyacrylamide gel electrophoresis (SDS-PAGE) and electro-transferred to nitrocellulose membranes. After blocking in TBST-5% non-fat milk powder for 1 hour, membranes were probed at 4°C for 24 hours with an anti-PGC-1α antibody from Santa Cruz Biotechnologies (Santa Cruz, CA), anti-GLUT1, anti-carnosine synthase (CS), or anti-lactate dehydrogenase (LDH) antibodies from Abcam (Cambridge, MA), normalized to an anti-β-actin primary monoclonal antibody from Sigma (St. Louis, MO) in TBST-1% non-fat milk powder overnight. Bound antibodies were detected by horseradish peroxidase-conjugated secondary antibodies from Sigma (St. Louis, MO) and by chemiluminescence using the ECL Plus Western Blotting Detection kit from GE Healthcare Life Sciences (Little Chalfont, Buckinghamshire, UK). Signal intensities were obtained by densitometry using ImageJ software (available from the NIH at http://rsbweb.nih.gov/ij/) by quantifying lane intensities followed by normalizing intensity with corresponding β-actin.

### Cell proliferation, migration and viability

Cell proliferation was assessed by measuring confluence-mask quantification via phase-contrast Incucyte ZOOM live content imaging and cell surveillance from Essen Bioscience (Ann Arbor, MI) in a 96-well plate. Cells were re-suspended in respective growth media with and without β-alanine at 100 mM, incubated and confluency was measured every hour for 48 hours. Cell migration was assessed by applying a standard scratch through confluent cells following treatment for 24 hours. The cells were then rinsed and the media replaced with corresponding treatments (control or β-alanine at 100 mM). Migration was determined by measuring the change in confluency for 24 hours following the initial wound. To assess changes in viability, cells were seeded overnight, and treated and incubated as described above for either 24 or 48 hours. The cells were then incubated for 1 hour in medium containing 10% WST-1 cell proliferation reagent from Roche (Indianapolis, IN) and fluorescence was measured using a Wallac Victor3V 1420 Multilabel Counter from PerkinElmer (Waltham, MA). To further investigate the effects of β-alanine on cell survival, cells were treated with varying concentrations (from 10 pM – 10 μM) of the antineoplastic Dox (in DMSO with final concentration = 0.1% for all Dox experiment groups including control) from Sigma (St. Louis, MO) with and without β-alanine at 100 mM for 24 hours. Treatment doses and duration were determined through pilot data for β-alanine and from previous observations for Dox [23].

### Statistical analyses

Metabolic measurements were analyzed by ANOVA with Tukey’s pair-wise comparisons for β-alanine compared with various controls. ATP concentration, cell migration, proliferation, immunoblotting, as well as flow cytometry and microscopy were analyzed using student’s *t*-test. Gene expression was quantified by relative expression using the ΔΔCp method, and analyzed using student’s t-test [18,20]. Cell viability was analyzed using analysis of variance (ANOVA) with Dunnett’s post hoc test. All data is represented as average ± standard deviation (SD) normalized to the control mean (control = 100) with *, **, and *** indicating p < 0.05, p < 0.01, and p < 0.001 statistical differences compared to control, respectively.

## Results

### β-alanine reduces glycolytic metabolism

**Figure 1:**
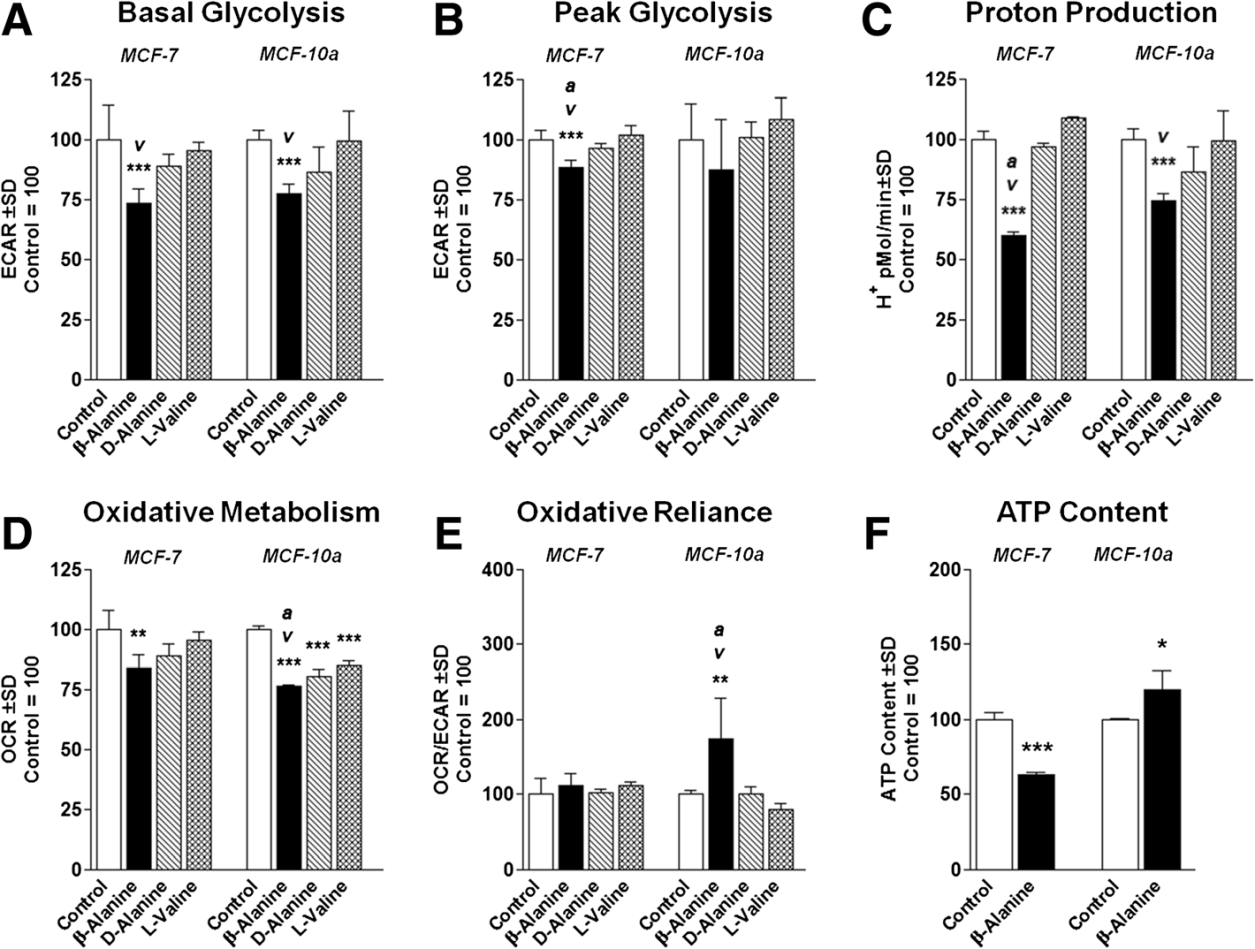
**β-Alanine alters glycolytic and oxidative metabolism in breast epithelial cells. (A)** Basal glycolytic metabolism reported as extracellular acidification rate (ECAR) following treatment of either MCF-7 or MCF-10a cells with β-Alanine, D-Alanine, or L-Valine at 100 mM for 24 hours. **(B)** Peak glycolytic metabolism (induced with oligomycin) for cells following treatment as described above. **(C)** Proton production rate during basal metabolism. **(D)** Basal oxidative metabolism reported as oxygen consumption rate (OCR) following treatment as described above. **(E)** Oxidative reliance expressed as a ratio of OCR:ECAR. **(F)** Cellular ATP production of both cell models following treatment as described above. NOTES: *indicates P < 0.05, **indicates P < 0.01, ***indicates P < 0.001 compared with control. *v* indicates P < 0.05 compared with L-Valine control. *a* indicates P < 0.05 compared with D-Alanine control.

To investigate the effects of β-alanine on aerobic glycolysis, we measured extracellular acidification rate (ECAR) following treatment for 24 hours. Both malignant (MCF-7) and non-malignant (MCF-10a) cells treated with β-alanine exhibited significantly reduced basal glycolysis compared with both true control and equivocal nitrogen load from 100 μM valine (Figure 1A). Additionally, β-alanine-treated MCF-7 cells demonstrated significantly reduced peak glycolytic capacities (peak glycolysis induced by oligomycin) compared with respective controls, but was unchanged in MCF-10a cells (Figure 1B). Similarly, both cell models exhibited significantly reduced cellular acidity following treatment with β-alanine compared with both true negative and valine control (Figure 1C).

To investigate the effects of β-alanine on oxidative metabolism, we measured oxygen consumption rate following treatment for 24 hours. Both MCF-7 and MCF-10a cells treated with β-alanine exhibited significantly reduced basal oxidative metabolism, however both D-alanine and L-valine controls were significantly reduced in the MCF-10a (Figure 1D). In addition to suppressed oxidative metabolism, MCF-10a cells exhibited increased oxidative reliance (expressed as a ratio of oxidative metabolism to glycolytic metabolism) illustrated in Figure 1E. Surprisingly, β-alanine-treated MCF-10a cells had significantly elevated total ATP content compared with their control, while MCF-7 ATP content was significantly reduced (Figure 1F).

### β-alanine alters glycolytic-related gene expression

**Figure 2:**
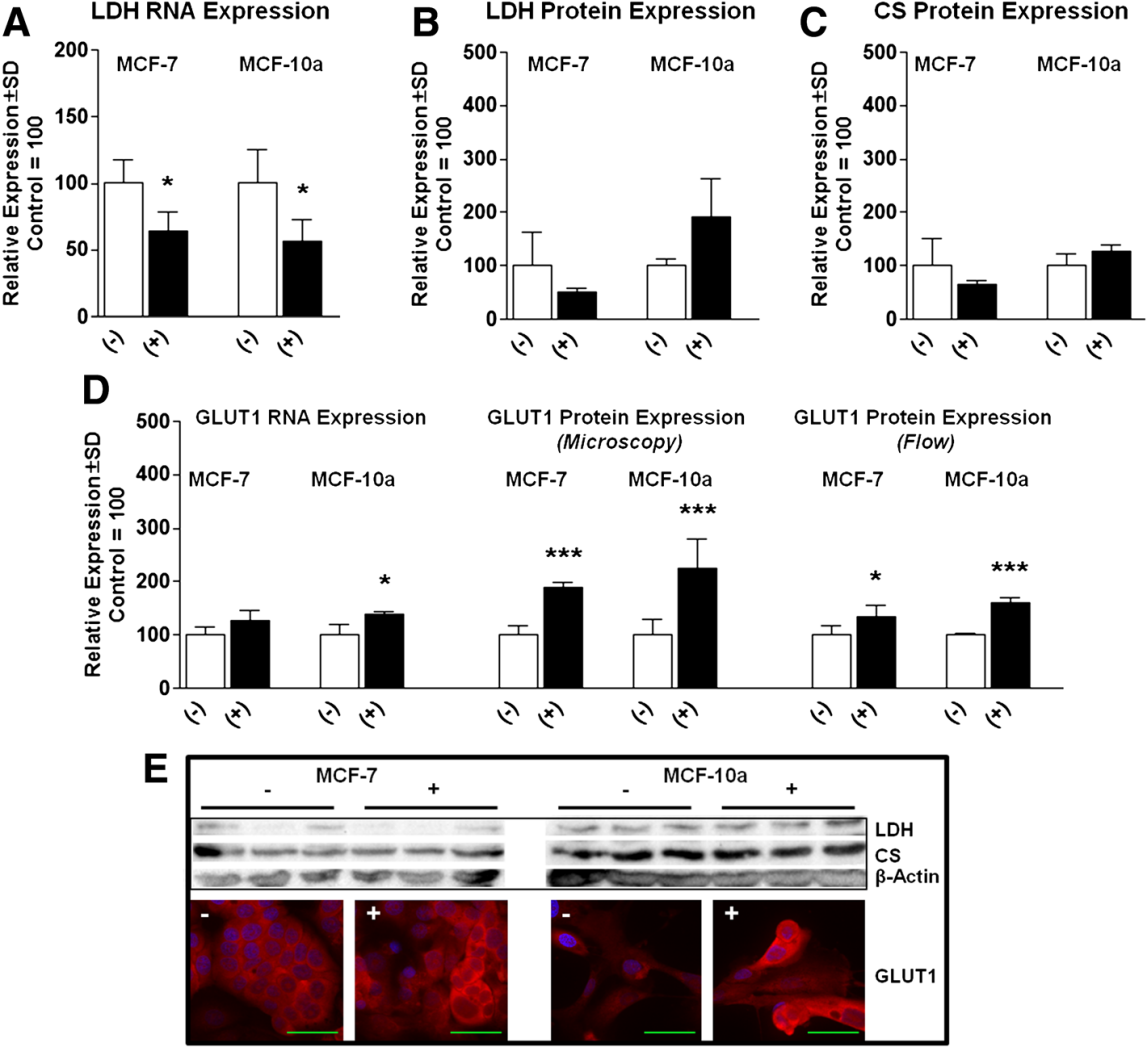
**β-Alanine alters glycolytic related gene expression in breast epithelial cells. (A)** Lactate dehydrogenase (LDH) RNA induction by qRT-PCR of MCF-7 and MCF-10a cells following treatment with either media control or 100 mM β-Alanine for 24 hours. **(B)** LDH protein expression following treatment as described above (representative blots displayed in **E**). **(C)** Carnosine synthase (CS) protein expression following treatment as described above (representative blots displayed in **E)**. **(D)** GLUT1 RNA induction (left) and GLUT1 protein expression quantified via confocal microscopy (center) or flow cytometry (right). Microscopy was quantified using 3 images and 7 representative cells/treatment. **(E)** Representative immunoblots and confocal microscopy images of MCF-7 cells (left) and MCF-10a cells (right) following treatment as described above (Blue = DAPI, Red = GLUT1, Green Size Bar = 50 μm). Images were taken using 63 × magnification with oil emersion. NOTES: *indicates P < 0.05, **indicates P < 0.01, ***indicates P < 0.001 compared with control.

To investigate the effects of β-alanine on gene expression related to aerobic glycolysis, we first measured LDH following treatment for 24 hours. Both MCF-7 and MCF-10a cells treated with β-alanine exhibited significantly reduced LDH mRNA levels while neither cell model exhibited significantly altered protein expression (Figure 2A and B, respectively). Because one potential mechanism of action of β-alanine could be through carnosine production, we measured Carnosine Synthase (CS) expression. CS was present in both cell models, but unaltered by β-alanine treatment (Figure 2C). In agreement with RNA levels, GLUT1 protein expression was significantly elevated in MCF-10a cells treated with β-alanine compared with control (measured by flow cytometry) which was verified by confocal microscopy (Figure 2D).

### β-alanine alters oxidative gene expression and mitochondrial content

**Figure 3:**
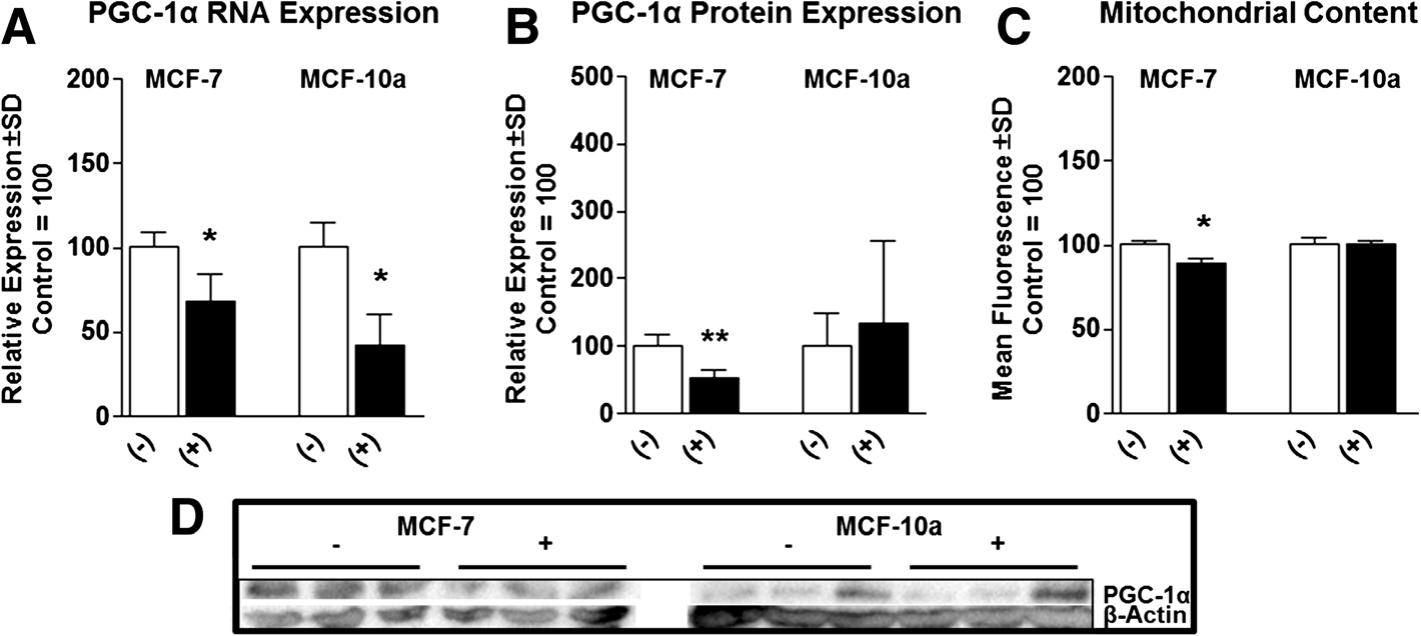
**β-Alanine selectively suppresses mitochondrial content and related gene expression in malignant breast epithelial cells. (A)** PGC-1α mRNA induction measured by qRT-PCR of MCF-7 and MCF-10a cells treated with and without β-Alanine for 24 hours. **(B)** PGC-1α protein expression following treatment as described above (representative blots displayed in **D)**. **(C)** Mitochondrial content measured by flow cytometry following Mitotracker green staining of MCF-7 and MCF-10a cells treated with and without β-Alanine for 24 hours. **(D)** Representative immunoblots of MCF-7 cells (left) and MCF-10a cells (right). NOTES: *indicates P < 0.05, **indicates P < 0.01, ***indicates P < 0.001 compared with control.

In order to evaluate the effects of β-alanine treatment on expression of genes involved in oxidative metabolism, we performed qRT-PCR following 24 hours treatment. Both β-alanine treated cell models demonstrated significantly suppressed RNA expression of PGC-1α (Figure 3A). Interestingly, PGC-1α protein expression was suppressed in MCF-7 cells but was unaltered in MCF-10a cells (Figure 3B). Consistent with PGC-1α protein expression, mitochondrial content was also suppressed in MCF-7 cells but was unaltered in MCF-10a cells (Figure 3C).

### β-alanine inhibits cellular proliferation without reducing viability and increases chemo-sensitivity

**Figure 4:**
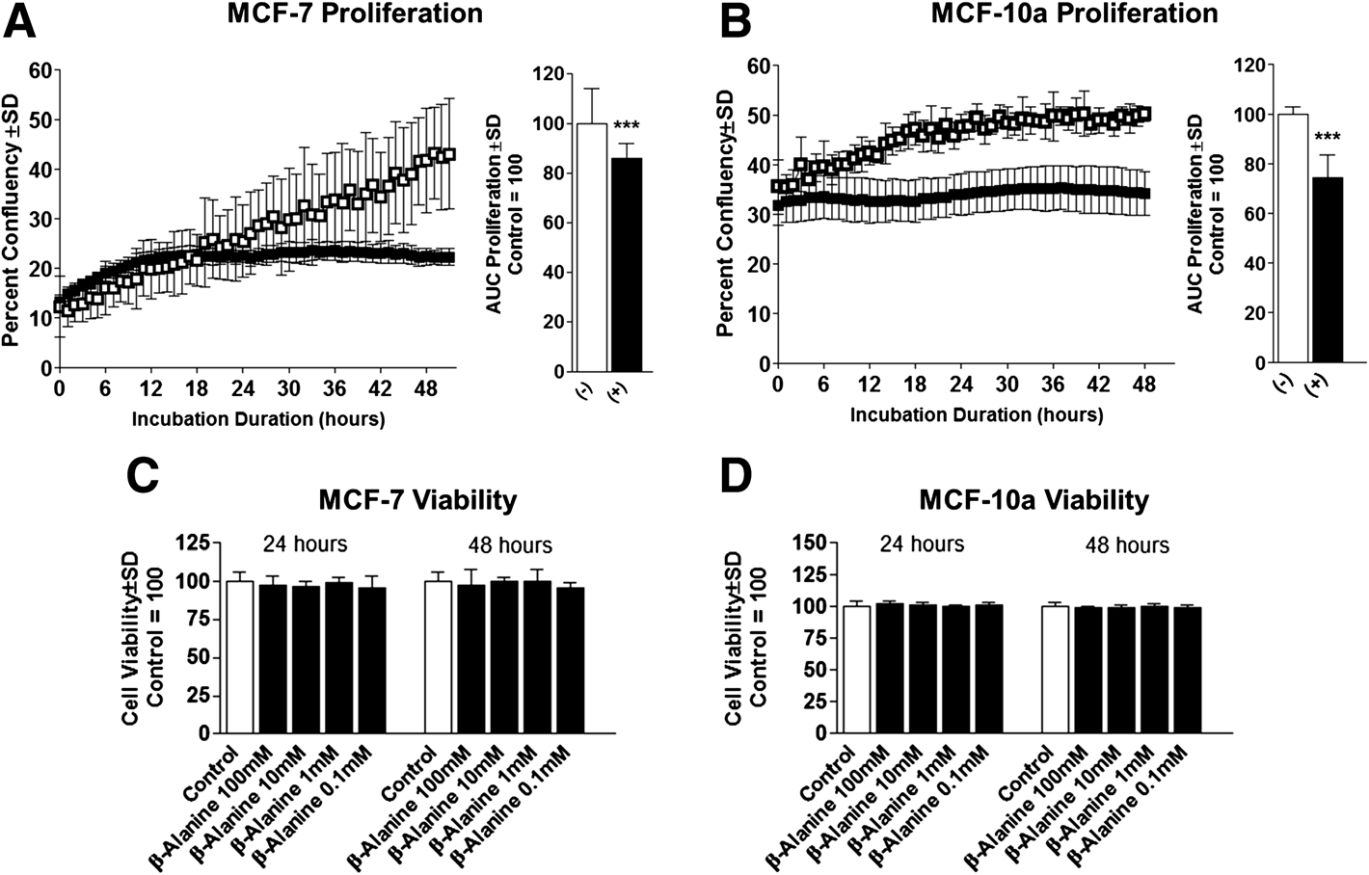
**β-Alanine suppresses breast epithelial cell proliferation without altering cell viability. (A)** Culture confluency of MCF-7 cells following treatment with control (−) or 100 mM β-Alanine (+) for 24 hours as measured by phase contrast live cell imaging. **(B)** Culture confluency of MCF-10a cells following treatment as described above. **(C)** Cell viability of MCF-7 cells treated as described above. **(D)** Cell viability of MCF-10a cells treated as described above. NOTES: *indicates P < 0.05, **indicates P < 0.01, ***indicates P < 0.001 compared with control.

**Figure 5:**
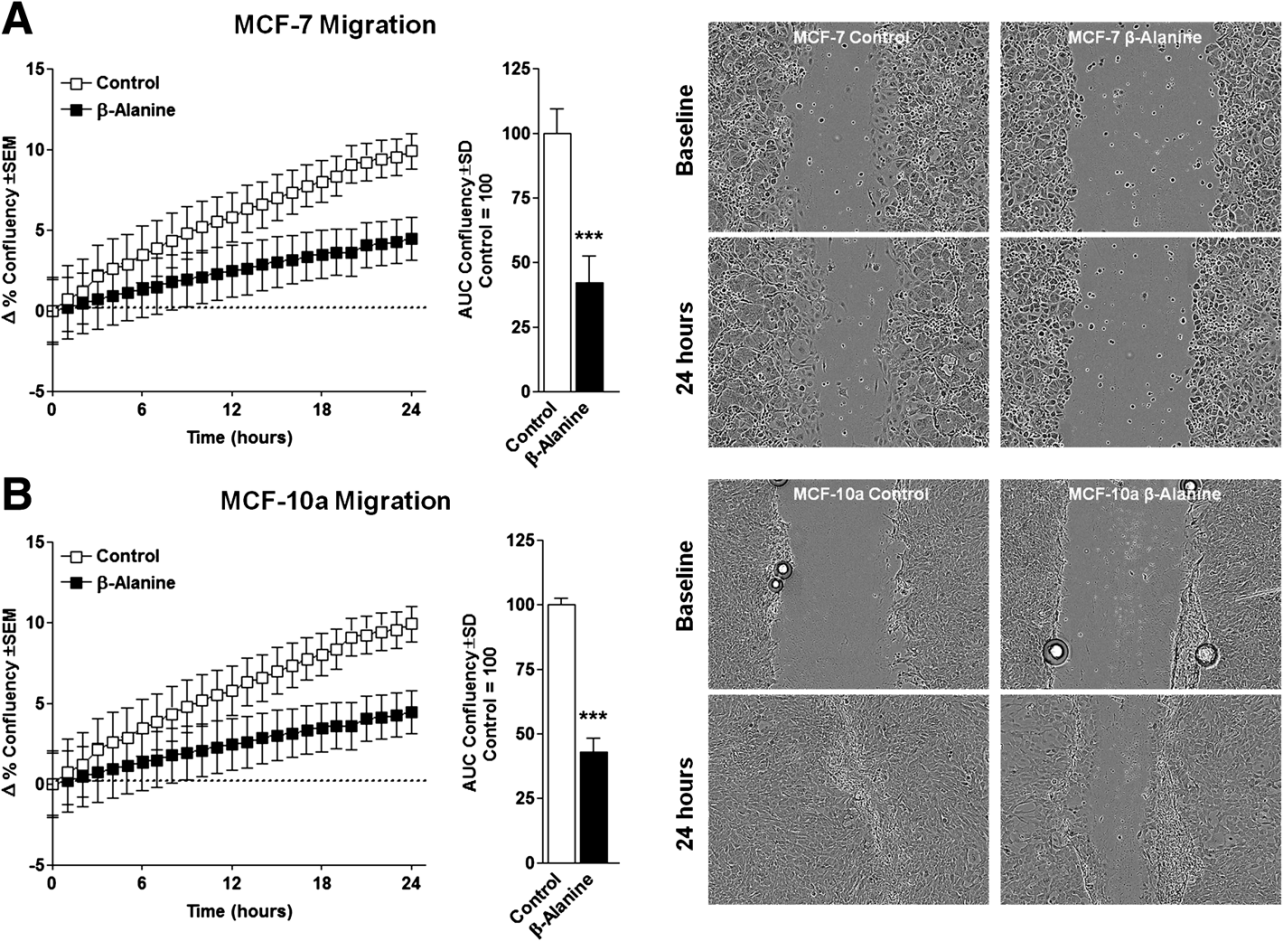
**β-Alanine suppresses breast epithelial cell migration. (A)** Migration measured in ∆ percent confluency analyzed as area under the curve (AUC) of MCF-7 cells following scratch and treatment with 100 mM β-Alanine measured hourly for 24 by phase contrast live cell imaging. Images on right show in baseline and 24 hour images of MCF-7 migration (top right). **(B)** Migration measured in ∆ percent confluency analyzed as area under the curve (AUC) of MCF-10a cells following scratch and treatment with 100 mM β-Alanine measured hourly for 24 by phase contrast live cell imaging. Images on right show in baseline and 24 hour images of MCF-10a migration (bottom right). Images were taken using 10 × magnification. NOTES: * indicates P < 0.05, ** indicates P < 0.01, *** indicates P < 0.001 compared with control.

**Figure 6:**
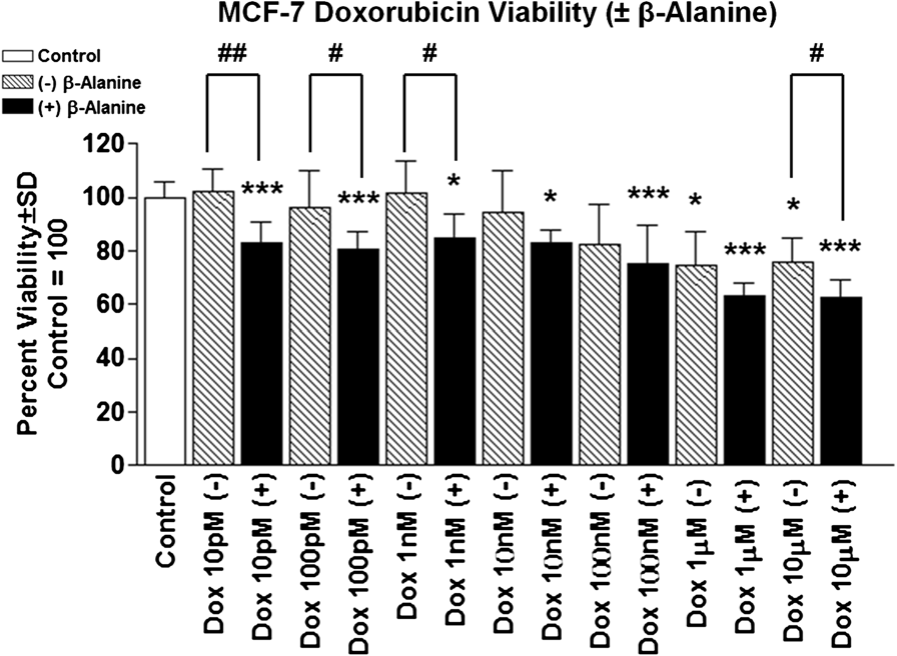
**β-Alanine enhances doxorubicin toxicity in malignant breast epithelial cells.** Cell viability of MCF-7 cells treated with increasing concentrations of Doxorubicin (Dox) in DMSO with and without 100 mM β-Alanine for 24 hours (final concentration for DMSO = 0.1%). NOTES: *indicates P < 0.05, **indicates P < 0.01, ***indicates P < 0.001 compared with control (above data = Dox-only and below data = Dox + β-Alanine). # indicates P < 0.05, ## indicates P < 0.01 for dose-comparisons of Dox treatment with and without β-Alanine.

In order to assess changes in cellular growth, we measured the effect of β-alanine on both cell models following treatment for either 24 or 48 hours. Proliferation of MCF-7 and MCF-10a was significantly suppressed following growth in media containing β-alanine compared with control (Figure 4A and B, respectively). Neither cell model experienced significant changes in cell viability at any of the tested doses (Figure 4C and D). To evaluate the effect β-alanine on cell migration, we measured cell motility via scratch-wound migration assay in a live cell imager taken hourly images for 48 hours. In both cell models, β-alanine treatment significantly reduced migration velocity compared with corresponding controls (Figure 5A and B). Because cellular uptake of Dox is significantly influenced by intra and extracellular pH, we investigated the effect of β-alanine on the efficacy of Dox. We treated MCF-7 cells with varying concentrations of Dox with and without β-alanine at 100 mM for 24 hours. Dox plus β-alanine treatment significantly reduced MCF-7 viability at all doses tested compared with DMSO control, an effect which was not seen in the Dox-only groups until high concentrations (1 μM -10 μM) (Figure 6). There were also significant reductions in MCF-7 viability at low doses of Dox with simultaneous β-alanine treatment. These observations suggest that β-alanine sensitizes cancer cells to Dox, thus improving the efficacy at lower concentrations.

## Discussion

Deregulated cellular energetics is now recognized as an emerging hallmark of cancer, central to most cancer phenotypes for reasons that remain elusive. In this report, we demonstrate that β-alanine, a commercially available product that exhibits intracellular buffering capacity, alters metabolic characteristics of malignant and non-malignant breast epithelial cells. Our experiments demonstrate that β-alanine can significantly reduce glycolytic metabolism with simultaneous reduction in corresponding cellular acidity. Additionally, we illustrate that β-alanine can selectively and significantly reduce oxidative metabolism by diminishing mitochondrial content in malignant cells. These results taken together demonstrate that β-alanine treatment can result in significantly suppressed basal metabolism; a potentially meaningful observation in cancer therapeutics.

Cells treated with β-alanine presented a favorable change in the microenvironment by increasing the alkalinity of the cellular microenvironment. These findings are consistent with previous observations that β-alanine consumption in humans increases blood pH and buffers lactate in anaerobically contracting muscle cells [10,24]. As mentioned above, extracellular pH plays a central role in regulation and activation of proteases used by malignant cells during migration and invasion [25,26].

Of interest are the effects that β-alanine or its metabolite carnosine may exhibit on tumor progression, which has been most heavily studied in neural cells [4]. Although oral administration of β-alanine increases carnosine content of select tissues (such as skeletal muscle), a concerning obstacle is that β-alanine treatment will only elicit anti-tumor effects in selective tissues that express carnosine synthase, suggesting that β-alanine’s benefits may be highly discriminatory [4,27]. While we demonstrate that both of the tested cell models express carnosine synthase, β-alanine treatment does not appear to alter carnosine synthase expression. This does not conclusively demonstrate that β-alanine-treated cells have equivocal carnosine content to untreated controls, but one might expect heightened carnosine synthase expression if it were a primary mechanism of action in β-alanine-treated cells.

It is also interesting that our observations show that β-alanine treatment can reduce metabolism and proliferation in breast epithelial cells (similar to previous results in glioma cells), without significantly altering cell viability [11-14]. A limitation of these observations is that β-alanine treated cells were tested in glucose-rich media, in which glucose was the primary substrate. If β-alanine inhibits glycolytic metabolism, then it is conceivable that β-alanine could reduce oxidative metabolism purely as a function of limited available substrate for oxidative metabolism. Hence, β-alanine may suppress oxidative metabolism by way of substrate blockade. In contrast to this hypothesis, we showed that β-alanine selectively reduces mitochondrial content in malignant MCF-7 cells by means of PGC-1α suppression; however this may only contribute some of the observed reductions in oxidative metabolism.

Also of great interest is the observation that β-alanine increases malignant breast cell sensitivity to Dox. Although not a clinical observation, our data further implicates β-alanine as a candidate for adjuvant therapy in malignancies such as breast cancer. Specifically, the observation that Dox plus β-alanine causes significantly reduced viability with extremely low concentrations of Dox (i.e. 10pM) suggesting combination therapy may lead to lower therapeutic concentrations of cytotoxic agents such as Dox. Although the precise mechanism(s) as to how β-alanine alters metabolism and chemotherapy sensitivity remains unknown, our data supports the hypothesis that alterations in cell energetics is a necessary component of β-alanine’s effect.

**Figure 7:**
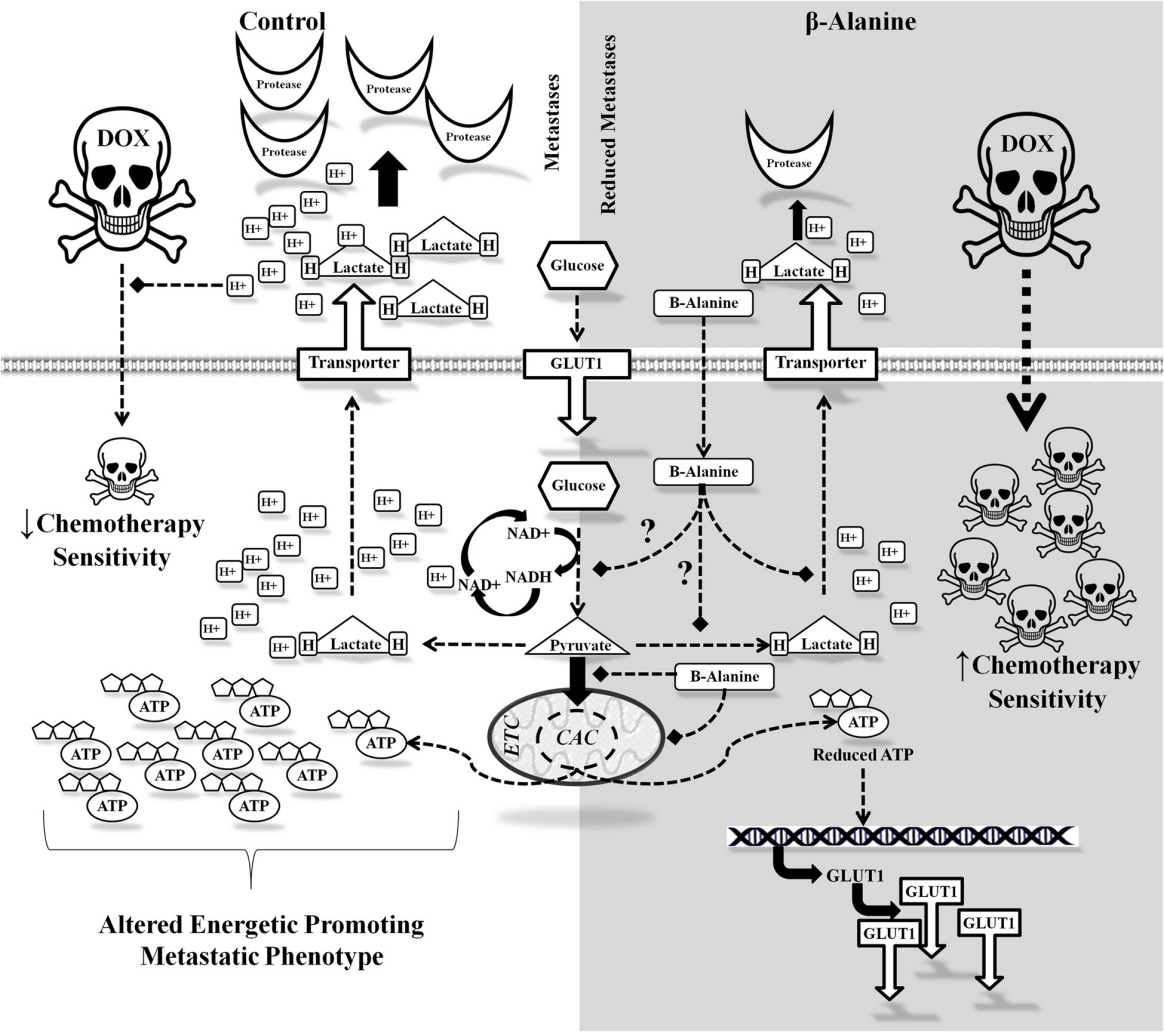
Summary of proposed potential mechanisms of action for intracellular β-alanine in the presence of chemotherapy Doxorubicin (Dox).

Figure 7 summarizes the proposed effects that β-alanine may exhibit on cell metabolism and metastatic phenotype. Briefly, glucose enters the cell via GLUT1, is metabolized through glycolysis to pyruvate, and is reduced to form lactate. Lactate along with protons are exported via monocarboxylate transporters decreasing extracellular pH leading to activation of proteases thereby increasing cell motility. Decreased extracellular pH in the microenvironment also leads to protonated Dox, thereby impeding cellular uptake and reducing efficacy (shown in Figure 7, left side) [7,8]. Conversely in β-alanine treated cells, glucose is taken up and metabolized (possibly at a lesser rate or magnitude than untreated cells), however both glycolytic and oxidative metabolism is reduced leading to reductions in ATP. Additionally, intracellular pH is increased due to suppression of anaerobic glycolysis, leading to reductions in lactate, proton production, and migration [6]. Increased extracellular pH leads to improved Dox transport thereby increasing efficacy and reducing the minimal therapeutic dose (shown in Figure 7, right side) [7,8]. It is also important to mention that we cannot rule out alternative mechanisms of action of β-alanine, or a possible role of carnosine resulting in suppressed malignant metabolic phenotype.

## Conclusion

Taken together, our results in combination with previous observations suggest that β-alanine may elicit several anti-tumor effects (possibly through carnosine biosynthesis), the mechanisms of which remain ill-defined and seemingly tissue-specific. Our observations support the need for further investigation into the mechanism(s) of action and specificity of β-alanine in tumor development and progression.

## Abbreviations

CS: Carnosine synthase; ECAR: Extracellular acidification rate; GLUT1: Glucose transporter 1; LDH: Lactate dehydrogenase; OCR: Oxygen consumption rate; PGC-1α: Peroxisome proliferator-activated receptor gamma coactivator 1 alpha; TBP: TATA binding protein.

## Competing interest

All authors and contributors declare no conflict of interest.

## Authors’ contribution

RAV Performed or oversaw all experiments, was primary author of manuscript, produced experimental design, performed statistical analyses and conceived the study. NPG assisted with manuscript writing and experimental protocols. RGS assisted in metabolic experiments. YLM and MAB assisted with various experimental protocols. RAV, MB, and KAT assisted with experimental design and manuscript production. All authors read and approved the final manuscript.
